# Clinical trials for Chagas disease: etiological and pathophysiological treatment

**DOI:** 10.3389/fmicb.2023.1295017

**Published:** 2023-12-15

**Authors:** Beatriz Matheus de Souza Gonzaga, Roberto Rodrigues Ferreira, Laura Lacerda Coelho, Anna Cristina C. Carvalho, Luciana Ribeiro Garzoni, Tania C. Araujo-Jorge

**Affiliations:** Laboratório de Inovações em Terapias, Ensino e Bioprodutos - Instituto Oswaldo Cruz, Fundação Oswaldo Cruz, Rio de Janeiro, Brazil

**Keywords:** Chagas disease, etiological treatment, pathophysiological treatment, clinical trial, *Trypanosoma cruzi*

## Abstract

Chagas disease (CD) is caused by the flagellate protozoan *Trypanosoma cruzi*. It is endemic in Latin America. Nowadays around 6 million people are affected worldwide, and 75 million are still at risk. CD has two evolutive phases, acute and chronic. The acute phase is mostly asymptomatic, or presenting unspecific symptoms which makes it hard to diagnose. At the chronic phase, patients can stay in the indeterminate form or develop cardiac and/or digestive manifestations. The two trypanocide drugs available for the treatment of CD are benznidazole (BZ) and nifurtimox (NFX), introduced in the clinic more than five decades ago. WHO recommends treatment for patients at the acute phase, at risk of congenital infection, for immunosuppressed patients and children with chronic infection. A high cure rate is seen at the CD acute phase but better treatment schemes still need to be investigated for the chronic phase. There are some limitations within the use of the trypanocide drugs, with side effects occurring in about 40% of the patients, that can lead patients to interrupt treatment. In addition, patients with advanced heart problems should not be treated with BZ. This is a neglected disease, discovered 114 years ago that still has no drug effective for their chronic phase. Multiple social economic and cultural barriers influence CD research. The high cost of the development of new drugs, in addition to the low economical return, results in the lack of investment. More economic support is required from governments and pharmaceutical companies on the development of more research for CD treatment. Two approaches stand out: repositioning and combination of drugs, witch drastically decrease the cost of this process, when compared to the development of a new drug. Here we discuss the progress of the clinical trials for the etiological and pathophysiological treatment for CD. In summary, more studies are needed to propose a new drug for CD. Therefore, BZ is still the best option for CD. The trials in course should clarify more about new treatment regimens, but it is already possible to indicate that dosage and time of treatment need to be adjusted.

## 1 Introduction

Chagas disease (CD) or American trypanosomiases, is named after the Brazilian scientist Carlos Chagas, who discovered this condition in 1909. It is caused by the flagellate protozoan *Trypanosoma cruzi* (Chagas, 1909). The last estimation sorted by the Pan-American Health Association (PAHO, 2023). It is estimated that 6–8 million people are infected with *T. cruzi* worldwide. 21 Latin American countries are mostly affected: Argentina, Belize, Bolivia (Plurinational State of), Brazil, Chile, Colombia, Costa Rica, Ecuador, El Salvador, French Guiana, Guatemala, Guyana, Honduras, Mexico, Nicaragua, Panama, Paraguay, Peru, Suriname, Uruguay, and Venezuela (Word Health Organization, 2023). Between 2000 and 2011 76.847 deaths were accounted for from tropical neglected diseases. CD was responsible for 76.7% of those deaths (Martins-Melo et al., 2016; DNDi, 2023).

Despite the control of vectorial transmission by *Triatoma infestants*, 75 million people still live in endemic areas with risk of infection (DNDi, 2023; Word Health Organization, 2023). In addition, oral transmission of *T. cruzi* is currently the main form of transmission in Brazil and is responsible for outbreaks of the disease in Amazon, Para, Santa Catarina, Bahia, Pernambuco and yet Colombia and Venezuela. In those areas, epidemiological context needs to me taken into consideration to avoid sub notification. It is estimated that for each acute notified case, 20/100 others could exist (Teixeira et al., 2001; Brasil, 2021). Between 2000 and 2010 more than 1,000 cases of CD were diagnosed in Brazil, 71% of those through oral infections (Shikanai-Yasuda and Carvalho, 2012; Araujo-Jorge et al., 2018). *T. cruzi* is a hemoflagellate protozoan that belongs to the *Trypanosomatidae* family (Levine et al., 1980). The parasite has four evolutionary forms: amastigote and epimastigote which are reproductive forms, and bloodstream (in vertebrates) and metacyclic (in the insect vectors) trypomastigote which are infectious but non-reproductive, forms (Brener, 1973; Ley et al., 2007). Infection begins during reduviid insects’ blood feed. When the excreta of the bug get in contact with the wound, metacyclic trypomastigotes penetrate host cells. Then, in the cytoplasm, they transform into amastigotes, divide through binary division and differentiate into bloodstream trypomastigotes. The rupture of the host cell membrane releases bloodstream trypomastigotes into the extracellular space to get into the blood, allowing them to get to any other location of the body and infect new cells (Brener, 1973; Burleigh and Woolsey, 2002).

Chagas disease has two clinical phases: acute CD (ACD) and chronic CD (CCD). Clinical symptoms in the acute phase begin 8–10 days after the infection, with short duration. It is characterized by acute inflammatory response, fever, presents unspecific symptoms and detectable parasitemia. In the acute phase the main cause of death is heart failure due to myocarditis, when the inoculum is high, or the host is immunosuppressed. However, in about 90% of the cases acute infection remains asymptomatic and is not noticed. The disease then enters remission beginning the chronic phase (Rassi et al., 2001, 2012; Echeverria and Morillo, 2019). The CCD is characterized by sub patent parasitemia, tissue parasitism and by a persistent low intensity inflammatory response. About 70% will remain in the asymptomatic “indeterminate” chronic clinical form, that has an indefinite duration and patients are seropositive but exhibit no detectable symptoms (Prata, 2001; Coura and Borges-Pereira, 2010; Echavarría et al., 2021). These are the most invisible cases, being discovered mainly when seropositive cases are detected among asymptomatic blood donors (Antunes et al., 2016): in São Paulo, Brazil, from 1996 to 2000, comparing mortality at a long term among 5,684 seronegative and 2,842 seropositive for asymptomatic indeterminate form of CCD, seropositive donors had a risk of death 17.9 times greater than seronegative donors, indicating the relevance of diagnostic strategies for both blood safety donation and CCD treatment. About 30% of the patients in the CCD will develop cardiac manifestations with complications such as: arrhythmia, thromboembolism, and heart failure. They are in the so-called chronic chagasic cardiomyopathy (CCC). Furthermore, 10% can present megacolon or megaesophagus, representing the digestive form (DCD). CCC has several pathological characteristics, such as thinning of the ventricular wall, changes in the size and shape of the heart, heart failure, atrophy with loss of myocardial fibers and the presence of dense and fibrous scar tissue (Bonney and Engman, 2008; Araujo-Jorge et al., 2022a). Cardiac manifestations of CD include abnormalities of the intraventricular conduction system, bradycardia, ventricular arrhythmias, sinus node dysfunction, heart failure, left ventricular aneurysms, and enlargement and dysfunction of the heart. Also, it’s possible to observe dilated coronary arteries with evidence of atherosclerosis. Heart failure and sudden death are the most common causes of death in patients with CD (Simões et al., 2007; Rassi et al., 2017).

The pathogenesis of chronic CCC is not completely understood (Rassi et al., 2017; Araujo-Jorge and Ferreira, 2022). Parasite persistence and inflammation with alteration of the host’s immune system can be implicated in the development of progressive heart damage caused by the infection (Coura and Borges-Pereira, 2010; Rossi et al., 2010). The symptoms presented and disease severity can be determined by the parasite–host combination, considering the relationship between the virulence of the *T. cruzi* strain and the genetic susceptibility of the individual (Lewis and Kelly, 2016; Zingales, 2018). Also, the intensity of the immune response will be directly related to the physiopathology of the disease (Gutierrez et al., 2009; Chevillard et al., 2018; Dantas-Pereira et al., 2021). It is possible to observe an exacerbated Th1 immune response with a pro-inflammatory profile, characterized by the release of interferon gamma (IFN-γ) and tumor necrosis factor alpha (TNF-α), in addition to decreased interleukin 10 (IL-10) release and suppression of cytokines related to the Th2 response, such as IL-4 (Ribeirão et al., 2000; Abel et al., 2001).

Cardiovascular commitment in the chronic phase can be classified according to the presence of ventricular dysfunction and symptoms of heart failure. Patients in stage A of CCC have cardiac alterations in electrocardiograms, which differ them from patients in the indeterminate phase of CD. In stage B1, patients are asymptomatic, but have alterations in the electrocardiogram and echocardiogram, presenting a left ventricle ejection fraction (LVEF) greater than 45%. In stage B2, patients present LVEF lower than 45%, but without presenting heart failure. Patients in stage C have ventricular dysfunction and symptoms of heart failure. Finally, in stage D, patients present symptoms of heart failure at rest that are resistant to treatment. Different prognosis and different mortality rates are related to the stages of CCC. The 5-year mortality rate for patients at stage D is 98%; 91% for patients at stage C, 45% for patients at stage B and only 13% for patients at stage A (Dias, 2015).

Mortality indicators for the subgroup of CD patients with heart failure are scarce. Systolic blood pressure, LVEF and the maximum rate of oxygen consumption are the most used for these patients (Mady et al., 1994; Theodoropoulos et al., 2008). The literature suggests LVEF alterations associated with the presence of movement abnormalities in segmental walls as the best indicator (Rassi et al., 2007).

The recommended treatment for heart failure is the same used in other etiologies, and relies on the use of beta blockers, angiotensin converting enzyme (ACE) inhibitors, diuretics, aldosterone antagonist and digoxin or the combination of hydralazine with isosorbide nitrate. In cases of arrhythmias the use of amiodarone may be indicated (Dias, 2015; CONITEC, 2018). The use of these drugs requires medical supervision with attention to some adverse effects that can be observed, such as postural hypotension that can be observed with the use of diuretics at long term; intoxication with digoxin, since this drug has a small therapeutic window. Hyperkalemia and loss of renal function may also be observed in patients using ACE inhibitors (Andrade et al., 2011; CONITEC, 2018).

Nowadays, two drugs are available for the trypanocide treatment of CD, introduced in the clinics for more than five decades (Peterson et al., 1979). Nifurtimox (NFX; 3-methyl-4-[59-nitrofurfurylideneamine] tetrahydro-4H-1,4-tiazine-1,1-dioxide) and benznidazole (BZ; N-benzyl-2- nitroimidazole acetamide), are nitroheterocyclic compounds with trypanocide activity (Coura and de Castro, 2002). During the 1980s, do Campo and Moreno (1984) intensively studied the mechanism of action of BZ and nifurtimox, concluding that the trypanocide activity was linked to the generation of free radicals and electrophilic metabolites (Do Campo and Moreno, 1984). NFX has its trypanocide activity associated with oxidative stress that occurs due its transformation into a nitro anion radical by the enzyme type II nitroreductase (NTR-II), with oxygen and ROS production (Do Campo and Stoppani, 1979). Furthermore, in BZ’s case, oxidative damage was not considered a key mechanism for its trypanocide action. Its activity was associated with the covalent binding of reduced metabolites to lipids, DNA and proteins (Polak and Richle, 1978; Diaz de Toranzo et al., 1988).

Chemotherapy against *T. cruzi* presents a cure rate of 90% for infants younger than 1 year old, 70% for patients at the acute phase of infection and 20% for patients at the chronic phase, as judged by the absence of parasitemia and negativation of serology (Coura and Borges-Pereira, 2012; Perez-Zetune et al., 2020). In some cases, parasites can persist even after treatment with BZ and patients still test positive for infection. So, seroconversion (lack of detectable *T. cruzi* antibodies in the serum) and parasite clearance by PCR can take many years. This is one of the difficulties in establishing a “proof of cure” in these patients (Chatelain, 2016; Kratz et al., 2018; Torrico et al., 2018).

Treatment with BZ is recommended for patients diagnosed in the acute phase, pregnant women, immunosuppressed patients, and all children with chronic infection, patients with indeterminate or digestive manifestations (PCDT-Chagas, CONITEC, 2018, PAHO, 2018). Adults up to 50 years old should be treated with BZ, as well as patients with heart problems in the initial phase, which should be decided in consensus with the patient. Additionally, in cases of advanced heart problems, treatment with BZ is not recommended, due to the controversy discussion of its benefits for these patients and lack of evidence from clinical trials to support this recommendation (CONITEC, 2018).

Both drugs available for CD present a series of unwanted side effects that occur in approximately 40% of treated patients (Lamas et al., 2006). Recent studies indicate a safer profile: Adverse drug reactions are common but were associated with low morbidity and were reversible upon discontinuation of drug treatment (Hasslocher-Moreno et al., 2012). Given that there were no fatal events, BZ treatment was safe. In older reviews, among the side effects it reported hypersensitivity, bone marrow depression and peripheral polyneuropathy (Cançado, 2002; Coura and de Castro, 2002). In many cases, the lack of medical follow-up can lead patients to interrupt treatment before completing the therapeutic scheme because of inadequate management of the adverse reactions (Viotti et al., 2009).

CD has an important economic impact globally, with a cost of about US$7–19 billion per year (Lee et al., 2013). The estimated health-care costs for CD are US$ 0.6 billion per year, with almost one-fifth of these costs occurring outside the endemic countries. In Brazil, the cost of hospitalization for chagasic cardiomyopathy with heart failure is higher than for non-chagasic patients with heart failure, and is estimated at US$ 467 per day (Abuhab et al., 2013).

Multiple cultural, economic, and diagnostic barriers limit the treatment of CD (Mills, 2020). Once high mortality rates are still observed in people with low incomes, reinforcing the social stigma of the disease, there is a low financial return forecast. Therefore, there is not much investment available for new drug deployment for CD (Pereiro, 2019; Ribeiro et al., 2020).

There are some challenges associated with the variety of unanswered questions about the disease mechanism and parasite–host interactions. Although, CD has been described 114 years ago, there is more to be elucidated about the factors that influence disease progression and the development of clinical manifestations, to identify possible therapeutic targets (Kratz, 2019).

An alternative in the search for more effective therapeutic schemes, is to use drug repositioning and combination (Araujo-Jorge et al., 2022b). Repositioning consists of using a drug already approved by health control agencies, to treat other diseases. Therefore, the compound has a pharmacokinetic profile already described and is safe for human use. This allows the research process to skip some phases, saving a lot of money and time. Using drug combination, reduction of BZ doses and treatment time can be achieved, which would help reduce side effects but maintain the trypanocide effect in the therapy scheme (Scarim et al., 2018).

According to DNDi, the ideal therapeutical scheme should consider parasitological cure for both acute and chronic phases; present no important side effects or teratogenic effects; should be effective in a short-term treatment, presented orally with possibility of dosage adjust based on patients’ age; should be financially accessible to patients (DNDi, 2019). The main limitations in evaluating treatment for chronic Chagas disease arise from the necessity of long-term follow-up, which usually lasts several decades. Moreover, the lack of standardization in tests to detect parasite elimination and of biomarkers for disease progression are also an impediment (Sales Junior et al., 2017).

In this review, we evaluate the progress of the clinical trials for physiopathological treatment for CD, including studies of new compounds, combination, and repositioning of drugs, highlighting their main findings, and discussing new treatment schemes for BZ and NFX.

## 2 Benznidazole

BZ is one of two drugs available for CD, included in the clinic in 1972 (Coura and Borges-Pereira, 2012). The need for high doses of BZ, due to its limited gastrointestinal absorption, in addition to the long-term treatment (2 months, but tuberculosis treatment is longer: 6 months) and the occurrence of side effects, are indicators of the need for new therapeutic schemes (Lamas et al., 2006; Viotti et al., 2009). According to clinicaltrials.gov fifteen trials evaluated the effect of BZ (Table 1), involving new treatment schemes, pediatric formulation, and combined therapy. Most of those studies are focused on patients in the chronic phase of CD and included outcomes for parasitological cure and cardiac improvement. Some of the clinical trials also investigate the BZ effect in patients with the indeterminate form of the disease. Treating patients with positive serology, even without the presence of symptoms, could lead to a negative seroconversion, preventing these patients from presenting any symptoms in the future.

**Table 1:** Clinical trials for etiological and pathophysiological treatment included in this study.

Clinical Trials	Register (clinicaltrials.gov)	Intervention	Target public	Patients included	Outcomes	Follow-up time	Results
Evaluation of Different Benznidazole Regimens for the Treatment of Chronic Chagas Disease. (MULTIBENZ).	#NCT03191162	BZ 300 mg/day for 60 days. 150 mg/day for 60 days. 400 mg/day for 15 days.	Patients with chronic CD (18 years and older).	Estimated enrollment: 240 patients.	Proportion of patients with negative parasitemia measured by PCR.	12 months after starting treatment.	No results posted.
CHICAMOCHA 3 - Equivalence of Usual Interventions for Trypanosomiasis (EQUITY) (CHICAMOCHA-3).	#NCT02369978	NFX 240 mg B.I.D for 60 days. 120 mg B.I.D for 120 days. BZ 300 mg B.I.D for 60 days. 150 mg B.I.D for 120 days.	Patients with serological evidence of Chagas infection and without clinical signs of dilated cardiomyopathy (20 to 65 years).	–	Proportion of participants with positive tests (qPCR) and with positive *T. cruzi* serology status. Mean change (before-after) in antibody readings as measured with ELISA serology. Reported adverse events.	12 to 18 months after starting treatment.	No results posted.
Etiologic Treatment With Benznidazole in Adult Patients With Chronic Chagas Disease. A Randomized Clinical Trial (TRAENA).	#NCT02386358	BZ 5 mg/kg/day for 60 days.	Patients with chronic CD (20 to 55 years).	---	Cardiovascular Mortality. Development of heart failure. Severe arrhythmias. Development of new permanent changes in the electrocardiogram. Clinical progression.	Until the date of first documented progression or date of death from any cause, up to 10 years of follow-up.	No results posted.
BENEFIT: Evaluation of the Use of Antiparasital Drug (Benznidazole) in the Treatment of Chronic Chagas’ Disease (BENEFIT).	#NCT00123916	BZ 300 mg/day for 40 or 80 days according to body weight.	Patients with serological evidence of Chagas infection presenting one or more indicators of heart disease (18–75 years).	2,854 patients were included.	Cardiovascular events: Death, Cardiac Arrest, Sustained Ventricular Tachycardia, Heart Failure, Pacemaker/ICD, Stroke/TIA or other Embolic Events, Cardiac Transplant. Progression of NYHA functional class. Evaluation of adverse events.	5 years.	BZ did not change the progression of cardiomyopathy in CD patients. BZ reduces circulating parasites in CD patients.
Randomized trial of efficacy of benznidazole in treatment of early *Trypanosoma cruzi* infection.	–	BZ 7.5 mg/kg/day for 60 days.	Children positive for *T. cruzi* antibodies (7–12 years).	130 children were included.	Disappearance of specific antibodies (negative seroconversion). Reduction of antibody titers on repeated serological tests.	3 years.	Treatment was safe and effective in negative seroconversion.
Population Pharmacokinetics in Benznidazole-treated Adults With Chronic With Chagas Disease (CINEBENZ).	#NCT01755403	BZ 2.5 mg/kg of BZ every 12 h for 60 days (300 mg/kg/day considering an average weight of 60 kg). 2.5 mg/kg/24 h for 60 days (150 mg/kg/day considering an average weight of 60 kg).	Patients with chronic CD (18 to 60 years).	39 patients were included.	Population pharmacokinetic parameters of BZ. Adverse events.	2 months.	The higher dose might lead to overexposure in most patients. The lower dose was able to achieve an accepted therapeutic range of BZ plasma concentrations.
Population Pharmacokinetics of Benznidazole in Children with Chagas Disease.	#NCT00699387	BZ 5 to 8 mg/kg/day for 60 days.	Children with CD (2 to 12 years).	40 children were included.	Population pharmacokinetics parameters of BZ. Adverse events.	2 months.	Lower BZ plasma concentrations were found in infants and children when compared to previously reported in adults treated with comparable mg/kg doses. Children have few adverse reactions to the drug when compared to adults.
Population Pharmacokinetics Study of Benznidazole in Children with Chagas’ Disease (Pop PK Chagas).	#NCT01549236	BZ 5.5 to 8.5 mg/Kg/day for 60 days.	Children with acute or early chronic indeterminate CD (1 day to 12 years).	81 children were included.	Pharmacokinetics parameters. Efficacy and safety.	2 months.	Lower BZ plasma concentrations were found in infants and children when compared to previously reported in adults treated with comparable mg/kg doses. Pediatric treatment was well tolerated, effective, and with mild adverse events.
Antibody drop in newborns congenitally infected by Trypanosoma cruzi treated with benznidazole.	–	BZ 5 mg/kg/day for 60 days. 7.5 mg/kg/day for 30 days.	Newborns with positive serological tests from a seropositive mother.	111 infected newborns.	Evaluate *T. cruzi* antibody titers using ELISA tests.	12 months.	In non-infected infants, mother’s antibodies disappeared quickly while in treated infected infants, the decrease of antibodies took longer and corresponded to the time of recovery.
Short-course Benznidazole Treatment to Reduce *Trypanosoma Cruzi* Parasitic Load in Women of Reproductive Age (BETTY).	#NCT03672487	BZ 150 mg/day for 30 days. 300 mg/day for 60 days.	*T. cruzi* seropositive women with a live birth during the postpartum period (13 years and older).	–	Frequency of positive PCR and the parasitic load measured by qPCR. Adverse events.	10 months.	No results posted.
New Therapies and Biomarkers for Chagas Infection (TESEO).	#NCT03981523	BZ 150 mg/day for 30 days. 150 mg/day for 90 days. 300 mg/day for 60 days. NFX 240 mg/day for 90 days. 480 mg/day for 30 days. 480 mg/day for 60 days.	Patients in the indeterminate form of CD (18 to 50 years).	450 patients were included.	Sustained parasitological clearance by qPCR. Changes over time in the blood parasitic load by qPCR. Changes over time in the conventional serology. Changes over time in non-conventional serology biomarkers.	3 years.	No results posted.
Study to Assess Bioequivalence of 30 and 120 mg Nifurtimox Tablets in Chronic Chagas’ Patients.	#NCT01927224	NFX 120 mg (one 120 mg tablet). 120 mg (four 30 mg tablets). 120 mg (as aqueous slurry in tap water).	Patients with chronic CD (18 to 45 years).	–	Pharmacokinetics parameters. Adverse events.	8 weeks.	No results posted.
Study Will Evaluate the Relative Bioavailability, Safety, and Tolerability of Single Doses of Nifurtimox 30 mg Tablets Exhibiting Different *In vitro* Dissolution Characteristics, and to Evaluate the Relative Bioavailability of Nifurtimox 30 mg and 120 mg.	#NCT03350295	NFX 120 mg (four 30 mg tablets). 30 mg (one 30 mg tablet). 120 mg (one 120 mg tablet).	Patients with chronic CD (18 to 45 years).	36 patients were included.	Pharmacokinetics parameters. Adverse events.	8 weeks.	NFX bioavailability was unaffected by tablet dissolution kinetics.
Study to Assess Bioequivalence of a New Nifurtimox Oral Tablet Formulation	#NCT03708133	NFX 120 mg new formulation. 120 mg current clinical formulation.	Patients with chronic CD (18 to 45 years).	–	Pharmacokinetics parameters. Adverse events.	6 months.	No results posted.
Prospective Study of a Pediatric Nifurtimox Formulation for Chagas’ Disease	#NCT02625974	NFX 10–20 mg/kg/day (aged <12 years) for 30 days plus 30 days with placebo. 10–20 mg/kg/day (aged <12 years) for 60 days. 8–10 mg/kg/day (aged ≥12 years) for 30 days plus 30 days with placebo. 8–10 mg/kg/day (aged ≥12 years) for 60 days.	Children with CD (up to 17 years).	330 children were included.	Negative seroconversion or seroreduction measured by two conventional ELISA serology tests. Presence of clinical signs/symptoms of chagas disease. Number of subjects with positive quantitative polymerase chain reaction (qPCR) results.	12 months.	NFX for 60 days achieved negative seroconversion and seroreduction. NFX for 30 days achieved negative seroconversion. Mild or moderate adverse events were observed and resolved without sequelae. The long-term follow-up (4 years) of the patients will provide important information about the progress of serological conversion in children treated with NFX.
Study to Assess the Food Effect on the Pharmacokinetics of Nifurtimox Tablets in Chronic Chagas’ Patients	#NCT02606864	NFX 120 mg (four 30 mg tablets).	Patients with chronic CD (18 to 45 years).	–	Pharmacokinetics parameters. Adverse events.	8 weeks.	No results posted.
Study to Assess the Food Effect on the Pharmacokinetics of Nifurtimox Tablets in Chronic Chagas’ Patients - Dietary Habits Study	#NCT03334838	NFX 120 mg (four 30 mg tablets). 240 mg (eight 30 mg tablets).	Patients with chronic CD (18 to 45 years).	24 patients were included.	Pharmacokinetics parameters. Adverse events.	8 weeks.	NFX bioavailability increased after a high-fat/high-calorie meal compared with a fasting state. NFX should be taken with food.
Study Of Nifurtimox Transfer into Breastmilk In Lactating Women With Chagas Disease (LACTNFX)	#NCT01744405	NFX 12 mg/kg/day for 30 days.	Lactating women with chronic CD.	10 women were included.	Nifurtimox concentration in breastmilk and in plasma. Adverse events.	30 days.	Low concentrations of NFX were found in breast milk. Breastfed babies presented normal clinical evaluation.
Clinical Trial For The Treatment Of Chronic Chagas Disease With Posaconazole And Benznidazole (CHAGASAZOL)	#NCT01162967	Posaconazole 800 mg for 60 days. 200 mg for 60 days. BZ 300 mg for 60 days.	Patients with chronic CD (18 to 45 years).	78 patients were included	Parasitological cure measured by a real time PCR. Safety and tolerability	12 months.	Posaconazole had antitrypanosomal activity in chronic CD patients. BZ group had less positive PCR results.
A Study of the Use of Oral Posaconazole (POS) in the Treatment of Asymptomatic Chronic Chagas Disease (P05267) (STOP CHAGAS)	#NCT01377480	Posaconazole • 800 mg for 60 days. • 800 mg for 60 days plus 200 mg of BZ for 60 days. • Placebo plus 200 mg of BZ for 60 days.	Adults with asymptomatic CD (18 to 50 years).	120 patients were included.	Parasitological cure measured by a real time PCR.	6 months.	Posaconazole had trypanostatic activity during treatment. In a long-term follow-up it was not effective for asymptomatic *T. cruzi* carriers. BZ in monotherapy was superior to posaconazole. A high rate of therapy discontinuation due to side effects were observed.
Proof-of-Concept Study of E1224 to Treat Adult Patients With Chagas Disease	#NCT01489228	E1224 4,000 mg for 8 weeks. 2000 mg for 8 weeks. 2,400 mg for 4 weeks plus placebo for 4 weeks. BZ 5 mg/kg/day for 60 days. Placebo for 8 weeks.	Patients with chronic indeterminate form of CD (18 to 50 years).	560 patients were included.	Proportion of patients with negative qPCR results. Incidence of negative serological conversion and changes in titers over time measured by conventional and non-conventional serologies. Pharmacokinetics parameters. Adverse events.	12 months.	E1224 treatment was safe and resulted in non-sustained parasite clearance for low-dose and short-dose regimens. BZ had sustained effect in 12-month follow-up.
BENDITA Benznidazole New Doses Improved Treatment and Associations (BENDITA)	#NCT03378661	BZ 300 mg/day for 8 weeks. 300 mg/day for 4 weeks. 300 mg/day for 2 weeks. 150 mg/day for 4 weeks. 150 mg/day for 4 weeks plus E1224. 300 mg once a week for 8 weeks plus E1224. 300 mg once a week for 8 weeks plus placebo.	Patients with chronic indeterminate form of CD (18 to 50 years).	210 patients were included.	Proportion of patients with negative qPCR results. Incidence of negative serological conversion and changes in titers over time measured by conventional and non-conventional serologies. Pharmacokinetics parameters. Adverse events.	12 months.	BZ had an effective antiparasitic response, independent of treatment duration, dose, or combination with E1224.
Pharmacokinetic Drug–Drug Interaction Study	#NCT03892213	BZ 2.5 mg/kg single dose at day 1 and 9 plus 2.5 mg/kg twice a day from day 12 until day 15. E1224 400 mg once a day from day 4 to day 6 followed by maintenance dose of 100 mg once a day from day 7 to day 15. *On day 9 and from day 12 to day 15, E1224 and BZ will be given concomitantly.	Patients with chronic CD (18 to 45 years).	Estimated enrollment: 28 patients.	Pharmacokinetics parameters. Adverse events. Clinical progression.	22 days.	No results posted.
Effects of Omega-3 Supplementation on the Cytokine and Lipid Profiles in Patients with Chronic Chagas Cardiomyopathy	#NCT01863576	Omega-3 5,500 mg of lipids, and 2000 mg of omega-3, 9 g of omega-3 per day for 8 weeks (5 capsules).	Patients with CCC (18 to 85 years).	42 patients were included.	Cytokine profile. Lipid profile. Anthropometric measures.	8 weeks.	Omega-3 supplementation decreased triglycerides and improved IL-10 concentrations, presenting a potential benefit for CCC patients.
Selenium Treatment and Chagasic Cardiopathy (STCC)	#NCT00875173	Sodium selenite 100 mcg for 1 year.	Patients with CCC (18 to 75 years).	66 patients were included.	Ejection fraction by echocardiography. Cardiac alterations by electrocardiography.	12 months.	Se treatment was safe for CCC patients. Se treatment did not improve cardiac function in CCC. A potential benefit of Se treatment was observed in the subgroup of patients at B2 stage.
Exercise Training in Chagas Cardiomyopathy	#NCT01006473	Exercise training 15-min warm-up, walking up to 30 min, followed by a 15-min cooling-down, three times a week for a total of 12 weeks (36 sessions).	Patients with CCC (30 to 65 years).	Estimated enrollment: 37 patients.	Functional capacity. Health related quality of life. BNP levels. Complications related to the exercise training.	12 weeks.	No results posted.
Cardiac Rehabilitation in Chagas Heart Failure	#NCT02516293	Exercise training • 30 min of aerobic exercise, 20 min of strength exercises and 10 min of stretching exercises three times per week for 8 months.	Patients with CCC (up to 18 years).	12 patients were included.	Functional Capacity. Muscle respiratory strength. Body composition. Cardiac function. Quality of life. Anthropometry.	8 months.	7 completed the 8-month follow-up period. Functional capacity improved after 4 months of cardiac rehabilitation. LVEF and respiratory strength improved after 8 months. Improvements in left ventricular diastolic pressure, respiratory strength, and quality of life were observed at the end of follow-up.
Exercise Training in Patients with Chagasic Heart Disease Without Ventricular Dysfunction (CH0660/10)	#NCT02295215	Exercise training • 5 min of stretching exercises, 40 min of aerobic exercises, 10 min of local strengthening exercises and 5 min of cool-down with stretching exercises, three sessions per week for 4 months.	Patients with CCC (25 to 60 years).	24 patients were included.	Sympathetic nerve activity. Autonomic control. Peak oxygen consumption. Skeletal muscle strength.	4 months.	Exercise training improved cardiac and peripheral autonomic function. Increased cardiac parasympathetic tone and increased oxidative metabolism of muscle fibers were observed. Exercise training decreased atrogin-1 gene expression.
Physical Exercise Program in Chronic Chagas Heart Disease (PEACH)	#NCT02517632	Exercise training • 30 min of aerobic exercise, 20 min of strength exercises and 10 min of stretching exercises three times per week for 8 months.	Patients with CCC (up to 18 years).	Estimated enrollment: 30 patients.	Functional capacity. Muscle respiratory strength. Body composition. Cardiac function. Quality of life. Microvascular reactivity. Body mass index.	6 months.	No results posted.
Amiodarone Against ICD Therapy in Chagas Cardiomyopathy for Primary Prevention of Death (CHAGASICS)	#NCT01722942	Amiodarone 600 mg/day for 10 days plus 200–400 mg/day (based on the therapeutic response on 24-h Holter monitoring) until the end of the study ventricular ICD implantation.	Patients with CCC (18 to 75 years).	Estimated enrollment: 1100 patients.	All-cause mortality. Cardiac mortality. Sudden cardiac death. Worsening heart failure warranting hospitalization. Need for cardiac stimulation in the ICD arm. Need for pacemaker implantation in the amiodarone therapy arm.	3 and half years.	No results posted.
A Trial Testing Amiodarone in Chagas Cardiomyopathy (ATTACH)	#NCT03193749	Amiodarone • 400 mg once a day for 10 days plus a maintenance dose of 200 mg once a day for at least 6 months, up to 24 months.	Patients with CCC (18 to 70 years).	Estimated enrollment: 200 patients.	Positive PCR for *Trypanosoma cruzi*	2 years.	No results posted.
Study to Evaluate Fexinidazole Dosing Regimens for the Treatment of Adult Patients With Chagas Disease	#NCT02498782	Fexinidazole 1800 mg for 2 weeks plus placebo to complete 8 weeks. • 1800 mg for 4 weeks plus placebo to complete 8 weeks. • 1800 mg for 8 weeks. • 1,200 mg for 2 weeks plus placebo to complete 8 weeks. • 1,200 mg for 4 weeks plus placebo to complete 8 weeks. • 1,200 mg for 8 weeks.	Patients with chronic indeterminate form of CD (18 to 50 years).	47 patients were included.	Proportion of patients with negative qPCR results. Parasite load. Serological response. Adverse events.	7 months.	Enrollment was interrupted after 4/47 patients presented grade 3 and 4 neutropenia. Rapid, sustained clearance of parasitemia was observed in all treated patients with available data. More studies are needed to elucidate the safety profile of fexinidazole.
Oral Fexinidazole Dosing Regimens for the Treatment of Adults with Chronic Indeterminate Chagas Disease (FEXI12)	#NCT03587766	Fexinidazole 600 mg for 10 days. 1,200 mg for 3 days. 600 mg for 3 days plus 1,200 mg for 4 days.	Patients with chronic indeterminate form of CD (18 to 60 years).	Estimated enrollment: 45 patients.	Pharmacokinetics parameters. Adverse events.	12 months.	No results posted.
Chagas Cardiomyopathy Bisoprolol Intervention Study: Charity	#NCT00323973	Bisoprolol 2.5 mg/day increased every 2 weeks to 5, 7.5 and 10 mg/day.	Patients with CCC (18 to 70 years).	Estimated enrollment: 500 patients.	Hospital admission caused by heart failure. Major adverse cardiovascular events. Heart failure worsening or mortality related with heart failure. Need for Implantable cardioverter-defibrillator (ICD), Cardiac resynchronization Therapy (CRT) or Pacemaker therapy (PM). Quality of life.	2 years.	No results posted.
Colchicine for Patients With Chagas’ Disease(B1 Stage) (COACH)	#NCT03704181	Colchicine 1 mg/day for 1 year	Patients with CCC (18 to 70 years).	Estimated enrollment: 60 patients.	Myocardial inflammation and fibrosis assessed by magnetic resonance imaging. Effect of colchicine on inflammatory markers such as interleukin-1, interleukin-6, interleukin-8, interleukin-10, TNF-α, IFN-γ. Proportion of patients with negative PCR-Real time results.	1 year.	No results posted.
Efficacy and Safety of Sacubitril/Valsartan Compared with Enalapril on Morbidity, Mortality, and NT-proBNP Change in Patients With CCC (PARACHUTE-HF)	#NCT04023227	Sacubitril/Valsartan Titrated doses from level 1 up to level 3 (100, 200 and 400 mg/day). Enalapril Titrated doses from level 1 up to level 3 (5, 10 and 20 mg/day).	Patients with CCC (up to 18 years).	Estimated enrollment: 900 patients.	Time to CV death, time to first heart failure hospitalization, relative change in NT-proBNP. Time to the first occurrence of cardiovascular events. Time to sudden death or resuscitated sudden cardiac arrest. Number of visits to an ER due to HF (where intravenous therapy is required). Number of days alive out of the hospital. Number of ventricular fibrillation or sustained ventricular tachycardia.	3 years.	No results posted.
Angiotensin Receptor-Neprilysin Inhibition in Chagas Cardiomyopathy With Reduced Ejection Fraction: ANSWER-HF. (ANSWER-HF)	#NCT04853758	Sacubitril/Valsartan • Titrated doses from level 1 up to level 3 (100, 200 and 400 mg/day) Enalapril. • Titrated doses from level 1 up to level 3 (5, 10 and 20 mg/day).	Patients with CCC (up to 18 years).	Estimated enrollment: 200 patients.	Change in LVEF. Cardiac worsening. Changes in New York Heart Association functional class. Evaluation of biomarkers - NT-proBNP, systemic cytokines, creatinine, urea, potassium, sodium.	6 months.	No results posted.
A Randomized Trial of Carvedilol in Chronic Chagas Cardiomyopathy	#NCT01557140	Enalapril Up-titrated to 40 mg/day and spironolactone 25 mg/day (all patients). Carvedilol 50 mg/day or placebo.	Patients with CCC (up to 18 years).	42 patients were included.	Changes in LVEF. Cardiac worsening. Changes in New York Heart Association functional class. Quality of life.	8 months.	Treatment with enalapril and spironolactone with subsequent addition of carvedilol were safe. Benefits in cardiac function and clinical status were observed.

The maximum dose used in the trials was 400 mg of BZ divided in two doses (clinicaltrials.gov #NCT03191162), and the lowest dosage used was 150 mg/day, tested in several trials (clinicaltrials.gov #NCT03191162, #NCT02369978, #NCT01755403). The time of treatment varies according to the proposed protocol. Mostly, when high doses were used, the time was shorter, staying between 15 and 30 days, while for small doses, time can get to 120 days of treatment (clinicaltrials.gov #NCT02369978).

The MULTIBENZ (clinicaltrials.gov #NCT03191162) is a phase II, randomized, double-blind, multicenter clinical trial. This study is in course to evaluate different BZ treatment schemes: (i) BZ 150 mg/day for 60 days, (ii) 400 mg/day for 15 days or (iii) 300 mg/day for 60 days. The outcomes to be evaluated are sustained parasitic load reduction measured by PCR, drug tolerability and pharmacokinetics parameters in a 12-month follow-up (Molina-Morant et al., 2020).

The EQUITY (clinicaltrials.gov #NCT02369978) is a randomized, blind, parallel-group trial to evaluate the trypanocide effect and safety of NFX and BZ in asymptomatic patients with *T. cruzi* positive serology. Participants were divided in: (i) 300 mg/day of BZ or 480 mg/day of NFX for 60 days (conventional scheme), (ii) 150 mg/day of BZ or 240 mg/day of NFX for 12 days, (iii) placebo treatment for each time of treatment. The primary outcome is the rate of positive PCR results (at least once for up to three), 12–18 months after randomization. The safety outcome evaluates moderate to severe adverse reactions, consistent blood marker abnormalities or treatment abandons. There are still no results available for this trial (Villar et al., 2019). Parasitological clearance verified through negative qPCR results and seroconversion, through the evaluation of *T. cruzi* serology status are the main outcomes used in the trials at the end of a 12 to 36 months follow-up.

Two of the trials registered in clinicaltrials.gov aimed to evaluate the effect of BZ treatment over cardiac alterations caused by the disease. The TRAENA clinical trial (clinicaltrials.gov #NCT02386358) was one of them. The registered outcomes were cardiovascular mortality, development of heart failure and severe arrhythmias with hemodynamic compromise, changes in the electrocardiogram, clinical progression and serological negativation. However, no results or publications were attached to this trial yet, despite specific comments on different CD treatment reviews (Paucar et al., 2016).

The BENEFIT (clinicaltrials.gov #NCT00123916) was the second trial with cardiovascular outcomes. The project started in 2005 and is one of the most important studies evaluating BZ treatment. This double-blind, placebo-controlled study recruited CCC patients from 54 study centers. The trial evaluated the effect of a fixed BZ dose of 300 mg for 40–80 days, and the time of treatment was adjusted according to the patient’s body weight. Their goal was to analyze the effect of BZ treatment over cardiac clinical progression signs such as: death, sustained ventricular tachycardia, new/worsening heart failure, stroke, or other embolic events. Also, they verified if BZ had effect over parasite burden, evaluated by qualitative and quantitative PCR, and the safety and tolerability of the treatment scheme proposed. The study ended in 2015, concluding that BZ did not change the progression of cardiomyopathy in chagasic patients. Although, treatment was able to reduce circulating parasites in these patients (Morillo et al., 2015). However, some of the coauthors of this important study contest this conclusion arguing that the final BENEFIT protocol did not follow the initial recommendations, thus leading to the inclusion of patients that could contribute to bias the results (Rassi et al., 2017). They stated that “several features of the BENEFIT trial raise some concerns and merit further discussion before concluding that benznidazole has no role in the treatment of patients with established CCC.” In addition, observing that “all components of the primary endpoint were, albeit not attaining statistical significance, less frequent in the benznidazole group than in the placebo group: patients receiving benznidazole had significantly fewer admissions to hospital for cardiovascular causes than those receiving placebo.” The authors discuss that “it is possible that the results of BENEFIT could become positive if admission to hospital was included in the composite endpoint,” as they suggested in the original protocol. “Keeping patients out of hospital is a major goal in the treatment of patients with CCC and, even as a post-hoc analysis, this finding may be informative.” Finally, these authors raised the question of whether the BENEFIT was underpowered, since the event rate in the placebo group was lower than expected (5.4% instead of 8% per year), so impacting in the BENEFIT trial power to detect significant differences in cardiovascular events between the BZ-treated and the placebo groups.

The discussions raised by these experts (Rassi et al., 2017) highlight the complexity of establishing clinical trial protocols for trypanocide drug effectivity testing: (Abel et al., 2001) the protocol must exclude patients with advanced heart disease or who already manifested a clinical condition that is a component of the primary endpoints of the study, as well as patients who are susceptible to reinfection, depending in the geographic area of recruitment; (Abras et al., 2022) the projection of the major outcome event rate in the placebo group should be sufficient to discriminate at least an effect of the intervention, attaining at least 20% relative risk reduction; (Abuhab et al., 2013) parasite-related outcomes should be used carefully (clearance of parasitemia indicated by PCR or disappearance of antibodies/ negative seroconversion). The results of conventional serological assays remain positive for years or even decades after successful therapy and the negative results of PCR assays after treatment are not reliable markers of cure. A negative PCR result does not necessarily rule out infection; it indicates only the absence of circulating *T. cruzi* DNA in the blood sample used for testing; (Alonso-Vega et al., 2021) even if the drug eliminates the parasite, the mechanism of disease progression may not be exclusively parasite-related. So, they defend that only cardiac and clinical alterations should be used as outcomes.

Whether adult patients with long lasting *T. cruzi* infection should be treated is an important discussion involving BZ treatment, even though the BENEFIT trial did not show evidence that BZ treatment affects the progression of cardiomyopathy in patients in the chronic phase with established cardiomyopathy (Morillo et al., 2015). Several studies point out evidence that supports the role of parasites on the progression of CCC and should be taken into consideration (Andrade et al., 2014). In addition, a non-randomized, non-placebo, controlled study of 2006, used 5 mg/kg/day of BZ (300 mg considering an average weight of 60 kg) for 30 days in adult patients with chronic CD. This study demonstrated reduction in the progression of CD with increase in negative seroconversion for patients with no heart failure (Viotti et al., 2006). Recent longitudinal studies focusing on large cohorts of asymptomatic indeterminate CCD patients indicate the relevance of BZ treatment to prevent CD progression: (Abel et al., 2001) in a study with 244 CCD blood donors, parasitemia was significantly reduced in the group previously treated with BZ, compared to the untreated group (Antunes et al., 2016); (Abras et al., 2022) 1,813 patients from 21 remote towns in Brazil, demonstrated that after 2 years of follow-up patients previously treated with BZ had significantly reduced parasitemia, a lower prevalence of markers of severe cardiomyopathy, and lower mortality after 2 years of follow-up (Cardoso et al., 2018); (Abuhab et al., 2013) in a retrospective cohort observational study including patients with CCD treated with BZ and compared to a group of non-treated patients matched for age, sex, region of origin, and the year of cohort entry, BZ treatment was associated with a decreased incidence of CCD progression and also with a decreased risk of cardiovascular events, indicating that BZ treatment for should be implemented into clinical practice managing the indeterminate form (Hasslocher-Moreno et al., 2021). All these results indicate the need for more clinical trials dealing with BZ and other trypanocide drugs.

Little is known about human BZ pharmacokinetics. Population pharmacokinetics is a very important tool to evaluate the effects of physiological factors over drug exposure. Therefore, a study was performed to characterize BZ pharmacokinetics in adult patients with CCD (clinicaltrials.gov #NCT01755403). Patients received 2.5 mg/kg of BZ every 12 h for 60 days. Data from simulations revealed that a dose of 2.5 mg/kg/12 h (300 mg/kg/day considering an average weight of 60 kg) might lead to overexposure in most patients. In this study, they also evaluated the use of a lower dose of 2.5 mg/kg/24 h (150 mg/kg/day considering an average weight of 60 kg), which achieved an accepted therapeutic range of BZ plasma concentrations of 3 to 6 mg/liter (Soy et al., 2015). Literature discusses the need for high BZ doses to achieve the desirable therapeutic responses, as a reflection of its low solubility in water (Lamas et al., 2006; Leonardi et al., 2009), in contrast, this study shows the reduction of BZ dose to 2.5 mg/kg/24 h should be recommended for most patients. Even though this group showed in a previous study that BZ concentrations might not be related to the appearance of serious drug effects (Pinazo et al., 2013), in this paper they indicated that such high drug concentrations are neither desirable nor needed to treat patients with CCD. Finally, these results reinforce the idea that the optimization of BZ is needed specially reducing BZ dose to treat adults with CCD (Soy et al., 2015).

Two studies evaluated the pharmacokinetic of BZ in children with CD treated with this drug. The first one tested a therapeutical scheme of 5 to 8 mg/kg/day for 60 days (clinicaltrials.gov #NCT00699387). Forty children from 2 to 12 years were included in the study (Altcheh et al., 2014). The second one (clinicaltrials.gov #NCT01549236) verified the safety and efficacy of BZ in children, infants, and neonates with CD (formulated in 100 mg tablets or 12.5 mg dispersible tablets). 81 children were divided into: newborns to 2 years and 2–12 years and treated with BZ 7.5 mg/kg/day in two daily doses for 60 days (Altcheh et al., 2023). Lower BZ plasma concentrations in infants and children were observed in comparison to what was previously reported in adults treated with comparable mg/kg doses. Also, children had few adverse reactions to the drug, indicating pediatric treatment was well tolerated and universally effective (Altcheh et al., 2014, 2023).

The understanding of the best pediatric scheme for BZ is a target of several clinical trials. A randomized, double-blind, placebo-controlled trial from 1995, treated schoolchildren aged 7–12 years, with 7.5 mg/kg/day for 60 days. They verified treatment was safe and led to negative seroconversion, evaluated through the disappearance of specific antibodies used to determine parasite clearance (Andrade et al., 1996).

Another study investigated parasite clearance through the drop of *T. cruzi* antibody titers. In this study, the authors compared the drop of antibodies in non-infected and congenitally infected newborns treated with BZ. Because of the lack of BZ pediatric formulation, 100 mg BZ tablets were ground up and reformulated in capsules of 8, 10, 13 and 15 mg. Newborns were divided into three groups: non-treated; treatment scheme A (2.5 mg/kg twice a day for 60 days) or treatment scheme B (7.5 mg/kg once a day for 30 days). The recovery was confirmed after 9 months of treatment in most infants and the decrease of *T. cruzi* antibodies does not depend on the treatment mode (Chippaux et al., 2010).

Congenital CD represents a big challenge in CD control, especially in non-endemic areas. This leads to an acute-phase disease in newborns, and many of them are asymptomatic. Therefore, all infants with missed congenital CD are at risk for a later development of CCD (Abras et al., 2017). BZ pediatric formulation is a huge innovation in CD treatment, allowing healthcare professionals to properly treat newborns and children, providing the right dose and duration of treatment. It is commercialized in an easy-to-use soluble tablet designed for infants and children up to 2 years of age and was included in the WHO List of Essential Medicines for Children in 2013. According to DNDi, pediatric BZ is in the phase of “registration and access” of drug development. At the moment, the drug is registered in Brazil, United States and Argentina, but there are still some endemic areas in the need for this new formulation (DNDi, 2021). In that case, physicians are forced to treat children with CD with improvise therapeutic options, such as fractionating the adult formulation, a faulty practice with risks and complications (Altcheh et al., 2023).

Studies suggest that congenital transmission does not occur in treated women, pregnant later in life. Since the level of parasitemia is a known risk factor for congenital transmission, BZ treatment can reduce parasite load and is recommended for these women. A short and low dose treatment scheme was proposed in a new clinical trial to investigate the occurrence of side effects and treatment compliance (clinicaltrials.gov #NCT03672487). Treatments proposed were: BZ 150 mg/day during 30 days and 300 mg/day during 60 days. This study is still in course (Cafferata et al., 2020).

Another limitation for treatment is the lack of consensus for how to treat patients with CD reactivation after immunosuppression due to heart transplant. Heart transplantation is a resource used for patients with advanced heart failure, that are refractory to treatment (Bocchi and Fiorelli, 2001; Gray et al., 2018; Moreira and Renan Cunha-Melo, 2020). CD reactivation is associated to the immunosuppressive protocols used to diminish the chance for rejection episodes after transplantation. The use of BZ for these patients is still in discussion since there is no scientific evidence to support this recommendation. Mostly, even with monitoring tests, BZ treatment is initiated only after clinical signs or symptoms similar to the acute phase appeared (Maldonado et al., 2004; Pinazo et al., 2011; Moreira and Renan Cunha-Melo, 2020). However, none controlled clinical trial was found to assess this issue.

Literature indicates that the use of BZ for CD treatment still needs improvement. Studies suggest that reduced doses with shorter time of treatment have good results on infection control, leading to negative seroconversion (Altcheh et al., 2014; Soy et al., 2015; Cortes-Serra et al., 2020). Treatment combination is a good alternative to reduce BZ dose (Coura, 2009). Since BZ has an essential trypanocide activity, some clinical trials evaluate its combination with other compounds. This matter will be addressed forward in this paper.

## 3 Nifurtimox

Nifurtimox was approved in 1965, at this time, the drug approval process was simpler and many preclinical and early clinical studies were not performed (Lang et al., 2023). Nowadays, eight studies are registered in clinicaltrials.gov.

Two of them compare the effect of different treatment schemes of both NFX and BZ. The first one is the EQUITY trial (clinicaltrials.gov #NCT02369978) mentioned earlier (Villar et al., 2019). The second one, is the TESEO study (clinicaltrials.gov #NCT03981523) an open-label, randomized, prospective clinical trial, with six treatment arms: (i) 150 mg of BZ twice a day for 60 days, (ii) 150 mg of BZ once a day for 30 days, (iii) 150 mg of BZ once a day for 90 days, (iv) 240 mg of NFX twice a day for 60 days, (v) 240 mg of NFX twice a day for 30 days, (vi) 240 mg once a day for 90 days. The outcome of this trial is sustained parasitological clearance evaluated by qPCR after a 36-months follow-up. This study is still ongoing (Alonso-Vega et al., 2021).

Bioavailability of NFX was evaluated in four clinical trials, all sponsored by Bayer. The first one (clinicaltrials.gov #NCT01927224) evaluate the bioequivalence, safety, and tolerability of a novel 30 mg tablet of NFX compared to the 120 mg tablet already commercialized for adults with CD. With the development of a 30 mg tablet NFX dose can be adjusted by age. The outcomes were the plasma concentration of NFX and the occurrence of adverse events. The second one (clinicaltrials.gov #NCT03350295) aimed to assess the bioavailability of three formulations of 30 mg tablets of NFX exhibiting different *in vitro* dissolution profiles. This study showed NFX bioavailability was unaffected by tablet dissolution kinetics (Stass et al., 2021). NFX pharmacokinetics profile would also be investigated. The third one (clinicaltrials.gov #NCT03708133) verified the bioequivalence of a new 120 mg tablet of NFX compared to the currently used in the Bayer pediatric development program. This new tablet should overcome the sensitivity to humidity problem observed earlier. Plasma concentrations of NFX and some pharmacokinetic parameters are the outcomes of this study. These studies are from 2014, 2018 and 2019 respectively, yet no registry of results was found. The last one had its results published in 2021. The CHICO trial (clinicaltrials.gov #NCT02625974) was a blinded, controlled study composed by 330 patients aged <18 years from 25 medical centers. Treatment schemes were: 10–20 mg/kg/day of NFX (aged <12 years) for 60 days; 8–10 mg/kg/day of NFX (aged ≥12 years) for 60 days or treated with one of NFX for 30 days added to placebo for 30 days. Overall serological response was lower for the 30 days treatment than for the 60 days. The frequency of *T. cruzi*-positive PCR results decreased from baseline levels for all treatment schemes. Adverse events were observed in 28.3% of patients treated for 60 days and 26.1% in patients treated for 30 days. The study concluded that NFX was well tolerated and safe for children with CD (Altcheh et al., 2021).

Finally, two studies, also sponsored by Bayer, assessed the food effect on the pharmacokinetics of NFX tablets. The 2015 study (clinicaltrials.gov #NCT02606864) investigated the effect of food on the absorption of the drug using the new 30 mg tablet in adults with CCD, after the ingestion of a high-calorie meal. The study outcome is evaluating plasma concentrations of NFX and adverse effects. The 2019 study (clinicaltrials.gov #NCT03334838) evaluated the bioavailability, safety, and tolerability of NFX in adults with CCC after a single oral dose of 120 mg administered under 3 types of fed conditions (low fat, dairy products, and high fat diets). The results indicate that NFX bioavailability increased after a high-fat/high-calorie meal when compared with a fasting state. Even though the type of diet affected bioavailability, the important conclusion was that NFX should be taken with food.

There are only a few trials that provide data of treatment efficacy in humans, pointing to its application in the acute phase, including congenital infection and early CCD (Pérez-Molina and Molina, 2018). Treatment with NFX is discouraged during breastfeeding, since there is no data evaluating if NFX is transferred into breast milk. Based on this, a study (clinicaltrials.gov #NCT01744405) was performed on lactating women with CCD to investigate if NFX is transferred into breast milk, and its safety for the newborns. It was concluded that there is only a limited drug transfer into breast milk, and no adverse reaction was observed in the breastfed infants. These results suggest that treatment for chronically infected women breastfeeding should be considered after delivery and the breastfeeding period (Moroni et al., 2019).

When used as a first-line treatment, NFX presents a high incidence of adverse effects (80.3–100%), the same safety profile can be observed when used after BZ intolerance. A high rate of treatment discontinuation was observed (18.4–43.8%) (Forsyth et al., 2016; Crespillo-Andújar et al., 2018). Literature has little data about NFX tolerance and safety, indicating that it should be more explored (Jackson et al., 2013). Also, there is no data of randomized clinical trials comparing BZ and NFZ; BZ is still generally preferred due to its better tolerability and tissue penetration, and adherence to treatment (Dias et al., 2016). There is no doubt about the need for a better understanding of the effects of NFX treatment schemes.

## 4 Posaconazole

Experimental studies with ergosterol inhibitors show their antiprotozoal activity, making them a promising alternative of treatment CCD (Urbina, 2010). Posaconazole is already approved for human use, to treat invasive fungal infection. Its pleiotropic effects have been described in CCD (Molina et al., 2015). In a murine model of acute CD, the drug was able to cure up to 90% of infected animals (Urbina et al., 1998). In addition, a study using a chronic model of CD, verified that posaconazole presented cure rates of up to 60% in infected animals, superior to what was observed for BZ treatment (Molina et al., 2000).

Two clinical trials with posaconazole are registered in clinicaltrials.gov. The first one (clinicaltrials.gov #NCT01162967) performed a randomized clinical trial to evaluate the efficacy and safety of posaconazole compared to BZ in adults in the chronic phase CD. The treatment scheme proposed was: high-dose of posaconazole (400 mg); low-dose of posaconazole (100 mg) and BZ (150 mg), all of them twice a day for 60 days. It was observed that 92% of the low-dose group and 81% of the high-dose group had positive PCR results. In contrast, only 38% of the patients treated with BZ had a positive PCR result (Molina et al., 2014). The STOP-CHAGAS trial (clinicaltrials.gov #NCT01377480) evaluated if posaconazole (400 mg) alone or combined with BZ (200 mg) had a superior outcome than BZ in monotherapy in eliminating circulating *T. cruzi* parasites. The study demonstrated that posaconazole had a trypanostatic activity during treatment, but it was not sustained in a long-term evaluation (Morillo et al., 2017). Both studies bring evidence that BZ, the standard of care, presents a better result than posaconazole.

Surprisingly, even with the promising results exhibited in literature, when tested in humans, posaconazole failed to achieve a sustained elimination of the parasites. In addition, in the second trial authors verified that treatment with BZ was discontinued by 32% of the patients, and the median time for interruption of the therapy was 40 days. Moreover, they observed a RT-PCR conversion of 90% in 30 days. This evidence supports other studies (Altcheh et al., 2014; Soy et al., 2015), that indicates a shorter time of treatment for BZ time should be considered.

## 5 Prodrug E1224

The prodrug E1224 is the first new chemical entity developed for Chagas disease in decades (Torrico et al., 2018). Prodrug E1224 metabolizes in ravuconazole, that inhibits *T. cruzi* ergosterol biosynthesis, essential for parasite growth and survival, becoming a promisor treatment for CCD (Urbina et al., 2003; Diniz et al., 2018).

Three studies were registered in clinicaltrials.gov. The first (clinicaltrials.gov #NCT01489228) was a proof-of-concept, double-blind, randomized clinical trial, investigating the safety and efficacy of different E1224 doses compared to BZ and placebo in adults with chronic indeterminate CD. Treatment schemes were: high-dose E1224 (8 weeks, with total dose 4,000 mg), low-dose E1224 (8 weeks - 2000 mg), short-dose E1224 (4 weeks – 2,400 mg + 4 weeks placebo), benznidazole (60 days - 5 mg/kg/day), or placebo (8 weeks). E1224 treatment resulted in non-sustained parasite clearance for low-dose and short-dose regimens. Otherwise, BZ had a rapid and sustained effect in a 12-month follow-up. They concluded E1224 was safe for individuals with chronic indeterminate CD (Torrico et al., 2018). The BENDITA trial (clinicaltrials.gov #NCT03378661) was a double-blind, multicenter, randomized study to assess the effect of the combination of BZ and E1224 in indeterminate CCD patients. Treatments proposed were: BZ 300 mg daily for 8 weeks, 4 weeks, or 2 weeks, BZ 150 mg daily for 4 weeks, BZ 150 mg daily for 4 weeks + E1224, BZ 300 mg once per week for 8 weeks +E1224, or placebo. In a 6-month follow-up, sustained parasitological clearance was observed in all treatment groups. Indicating BZ had a good effect regardless of treatment duration, dose, or combination with E1224 (Torrico et al., 2021). Finally, a pharmacokinetics study (clinicaltrials.gov #NCT03892213) is in course to determine whether BZ and E1224 should be administered concomitantly in patients with CD. The aim of this study is to assess cross interactions of these two compounds. There are no results available yet.

In the BENDITA study, 7% of the patients in long-term or high-dose treatment schemes had adverse events that led to treatment discontinuation. No adverse events leading to treatment discontinuation were observed in patients treated with benznidazole 300 mg daily for 2 weeks or placebo, adding evidence to the need for reduced or shorter BZ treatment schemes, to improve tolerability and adherence to treatment.

## 6 Natural compounds

Supplementation with natural compounds is discussed as an alternative for CD treatment (Jelicks et al., 2011; Maldonado et al., 2021). Literature indicates that omega-3 has important effects over treatment for cardiovascular diseases, reducing the occurrence of fatal events and hospital admissions (Nodari et al., 2011). A single-center double-blind clinical trial (clinicaltrials.gov #NCT01863576) was performed to investigate the effect of omega-3 supplementation in CCC patients. Patients received omega-3 PUFAs capsules (1.8 g EPA and 1.2 g DHA) or placebo (corn oil) for 8 weeks. They verified supplementation effects over the lipid profile, proinflammatory and anti-inflammatory cytokine levels. Improvements in serum triglycerides and IL-10 levels were observed, indicating that omega-3 could be a new coadjutant strategy to treat patients with CCC (Silva et al., 2017).

Studies also describe selenium treatment as a promising option for cardiovascular diseases. A study indicated that patients with severe cases of CD myocardiopathy presented decreased selenium levels (Rivera et al., 2002). The STCC trial (clinicaltrials.gov #NCT00875173) was created to evaluate the effect of selenium treatment on preventing cardiac disease progression when compared to placebo. Sixty-six patients in B1 or B2 stages of CCC were included and treated for 1 year with 100 mcg/day sodium selenite. Selenium treatment was safe, and no adverse effects were noticed. Treatment did not improve cardiac function evaluated through LVEF measures. Although, a potential beneficial effect of selenium was verified in patients at B2 stage, and more studies are necessary to explore different conditions, such as increased selenium dosage, associations with other supplements and longer follow-up time (Holanda et al., 2021).

## 7 Exercise training

Several benefits have been described for exercise training over heart failure (Batista et al., 2009; Sadek et al., 2022). Four trials are registered in clinicaltrials.gov to investigate this effect on CD patients. In 2007 the first trial (clinicaltrials.gov #NCT01006473) was initiated, to verify if exercise training could improve functional capacity, quality of life, and reduce brain natriuretic peptide levels in patients with CD to accompany for 12 weeks in 36 sessions. Yet, no result attached to this study.

In 2013 two clinical trials started. One of them (clinicaltrials.gov #NCT02516293) evaluated the effect of a three times per week, 60 min exercise session for 8 months. They aimed to assess functional capacity, muscle respiratory strength and body composition. Also, cardiac function, biomarkers and quality of life were included as outcomes. Seven of 12 patients completed the 8-month follow-up period. Exercise led to improvement of function after 4 months of cardiac rehabilitation, and LVEF and respiratory strength improved after 8 months. Also, improvements in left ventricular diastolic pressure, respiratory strength, and quality of life were observed at the end of follow-up (Mediano et al., 2016). The other 2013 trial (clinicaltrials.gov #NCT02295215) investigated the effect of exercise training in patients with subclinical CCC. Exercise training had beneficial effects for patients, such as: improved cardiac and peripheral autonomic function, increased cardiac parasympathetic tone, increased in oxidative metabolism of muscle fibers, and decreased atrogin-1 gene expression (Sarmento et al., 2021).

In 2015 the PEACH trial (clinicaltrials.gov # NCT02517632) was initiated. This single-center randomized study aimed to evaluate the effect of exercise training over functional capacity, cardiac function, quality of life, and biomarkers in CD patients with heart commitment (Mendes et al., 2016). Also, no result could be found for this trial. More clinical evidence is needed to discuss the use of exercise training for CD patients. Two clinical trials bring results of the effect of exercise training, but are insufficient to conclude about the benefits of this intervention over functional capacity and improvement of quality of life of patients.

## 8 Amiodarone

Amiodarone is an antiarrhythmic used in the clinic as treatment of choice for patients with sustained ventricular tachycardia, and for patients with myocardial dysfunction (Scanavacca et al., 2002). Also, studies indicate that amiodarone improves survival in patients with high risk of arrhythmic death (Rassi et al., 1995, 2001). Since there is no clinical evidence of the effect of BZ and NFX over cardiovascular alterations, treatment of heart failure and arrhythmias should be explored in CD. The first amiodarone trial (clinicaltrials.gov #NCT01722942) aimed to compare the efficacy of the implantable cardioverter defibrillator (ICD) with the use of amiodarone in patients with CCC and non-sustained ventricular tachycardia. The outcomes were: all-cause mortality, cardiac mortality, worsening of heart failure needed for pacemaker implantation (Martinelli et al., 2013). The ATTACH trial (clinicaltrials.gov #NCT03193749) was designed to assess the effect of amiodarone in patients with mild-to-moderate CCC. Patients will receive 400 mg of amiodarone hydrochloride once a day for 10 days plus 200 mg once a day for at least 6 months. The outcomes are PCR results for *T. cruzi* and clinical events as all cause death and hospitalization for cardiovascular causes. None of the trials had results attached to it.

## 9 Fexinidazole

Fexinidazole is a 2-substituted 5-nitroimidazole, antiprotozoal drug, candidate to treat sleeping sickness. Fexinidazole presents a promising safety and efficacy profile shown by preclinical studies (Torreele et al., 2010; Bahia et al., 2012). A double-blind, randomized, placebo-controlled trial (clinicaltrials.gov #NCT02498782) tested the effect of fexinidazole in adults with the chronic indeterminate form of CD. Treatment schemes were 1,200 or 1800 mg/day of fexinidazole for 2, 4, or 8 weeks or placebo. Patients’ enrollment was interrupted after some patients presented neutropenia, and treatment interrupted in all patients with less than 2 weeks. A sustained clearance of parasitemia could be observed in all treated patients with available data but additional exposure-response analysis are needed to clarify if low dosages of fexinidazole may be safer and effective (Torrico et al., 2023). Another trial (clinicaltrials.gov #NCT03587766) was created to assess the effect of low doses (600 and 1,200 mg) and short-term treatment of fexinidazole (3–10 days) in adult patients with chronic indeterminate CD. The outcomes for this study are Incidence and severity of adverse events and pharmacokinetics parameters. There are still no results for this trial.

## 10 Bisoprolol

Bisoprolol is a selective beta-1 selective blocker with the highest selectivity for this receptor, used to treat hypertension, chronic heart failure and angina pectoris. Literature shows that beta-blockers can reduce morbidity and mortality while tested in congestive heart failure (Levy et al., 1998). In addition, congestive heart failure caused by CD responds to treatment with diuretics and vasodilators (Hagar and Rahimtoola, 1995). The CHARITY trial (clinicaltrials.gov #NCT00323973) is a randomized, double-blind, placebo-controlled study, initiated to verify the effect of bisoprolol (10 mg) over cardiovascular mortality, hospital readmission due to progressive heart failure and functional status in patients with heart failure secondary to CD (Quiros et al., 2006). There is still no result for this study.

## 11 Colchicine

Colchicine is an anti-inflammatory drug commonly used to treat gout. Literature describes colchicine protective effect on myocardium, with decreased interstitial fibrosis and attenuated inflammation. CD is considered an inflammatory heart disease, and the use of colchicine presents an alternative to minimize myocardial damage and improve clinical outcomes (Niel and Scherrmann, 2006; Fernandes et al., 2012). A clinical study (clinicaltrials.gov #NCT03704181) aimed to assess the effect of colchicine on myocardial inflammation, inflammatory markers and PCR for *T. cruzi* on CD patients at B1 stage of CCC in a 1-year follow-up time. There is no result attached to this trial.

## 12 Sacubitril + valsartan

The drug combination sacubitril + valsartan is indicated for adult patients with chronic heart failure with reduced LVEF. The PARADIGM-HF study compared the effect of treatment with sacubitril + valsartan to ACE inhibitors and observed a 20% reduction in mortality. Reduced risk of cardiovascular death or hospitalization for heart failure was also related to sacubitril + valsartan treatment (Vardeny et al., 2016).

Based on this evidence two clinical trials were created to evaluate the effect of these drugs on CD patients. The multicenter PARACHUTE-HF trial (clinicaltrials.gov #NCT04023227) was designed to evaluate the effect of sacubitril/valsartan 200 mg compared with enalapril 10 mg in patients with CCC. The outcomes of the study all-cause mortality, sudden death, time to first heart failure hospitalization and changes in NT-proBNP levels in a 12–36 weeks follow-up. No result was found for this trial. The ANSWER-HF trial (clinicaltrials.gov #NCT04853758) is a randomized, single-center, double-blind, controlled study to evaluate the benefit of sacubitril/valsartan treatment for 6 months compared with enalapril in patients with heart failure due to CCC with reduced ejection fraction. The outcomes are changes of left ventricular ejection fraction, ventricular arrhythmias; functional capacity and ventricular remodeling. No result was found for both trials.

## 13 Carvedilol

Carvedilol is a non-selective beta blocker used in the clinic for severe congestive heart failure and arterial hypertension. A double-blind, placebo-controlled, randomized trial (clinicaltrials.gov #NCT01557140) was performed to investigate the safety profiles and efficacy of renin-angiotensin system inhibitors and beta-blockers in CCC patients. In the first phase all patients received enalapril (20 mg) and spironolactone (25 mg). Subsequently, patients were randomly divided in two groups: carvedilol (25 mg) or placebo. Treatment was safe and well tolerated, associated with benefits in cardiac function, with reductions on cardiothoracic index and BNP levels (Botoni et al., 2007).

## 14 Conclusion

This review covered clinical trials for etiological and pathophysiological treatment of CD, to provide an overview of the scientific evidence available. The studies regarding treatment options for CD have advanced a lot in the past years. However, there is still much work to be done. Before discussing any of the trials in course, the standardization of diagnostic methods used as outcomes needs to be prioritized, to improve the permit a proper comparison between the results obtained in the trials. This will allow the scientific community to determine the next steps to be followed. Another limitation is the long time necessary to assess serological reversion, the most used outcome in the studies. Moreover, research on effectible biomarkers accessible biomarkers for early detection of treatment efficacy is needed.

Some discrepancies can be found between clinical trials and observational studies, showing the complexities and controversies of science. Based on the results available for registered clinical trials, BZ is still the best choice for the treatment for CD. Nonetheless, clinical evidence indicates a reduced dose in a short-term treatment can be effective for many cases, diminishing adverse effects, though improving therapy adherence. In conclusion, the treatment scheme currently used for BZ needs to be reviewed.

## Author contributions

BG: Conceptualization, Writing – original draft, Writing – review & editing. RF: Conceptualization, Writing – original draft, Writing – review & editing. LC: Conceptualization, Writing – review & editing. AC: Conceptualization, Writing – review & editing. LG: Supervision, Writing – review & editing. TA-J: Conceptualization, Supervision, Writing – original draft, Writing – review & editing.
